# A Novel Function of Apolipoprotein E: Upregulation of ATP-Binding Cassette Transporter A1 Expression

**DOI:** 10.1371/journal.pone.0021453

**Published:** 2011-07-11

**Authors:** Yanfeng Zhao, Xinping Chen, Hong Yang, Lichun Zhou, Emmanuel U. Okoro, Zhongmao Guo

**Affiliations:** 1 Department of Physiology, Wuhan University School of Basic Medical Science, Wuhan, People's Republic of China; 2 Department of Physiology, Meharry Medical College, Nashville, Tennessee, United States of America; University of South Florida, United States of America

## Abstract

Despite the well known importance of apolipoprotein (Apo) E in cholesterol efflux, the effect of ApoE on the expression of ATP-binding cassette transporter A1 (ABCA1) has never been investigated. The objective of this study was to determine the effect of ApoE on ApoB-carrying lipoprotein-induced expression of ABCA1, a protein that mediates cholesterol efflux. Our data demonstrate that ApoB-carrying lipoproteins obtained from both wild-type and ApoE knockout mice induced ApoAI-mediated cholesterol efflux in mouse macrophages, which was associated with an enhanced ABCA1 promoter activity, and an increased ABCA1 mRNA and protein expression. In addition, these lipoproteins increased the level of phosphorylated specificity protein 1 (Sp1) and the amount of Sp1 bound to the ABCA1 promoter. However, all these inductions were significantly diminished in cells treated with ApoE-free lipoproteins, when compared to those treated with wild-type lipoproteins. Enrichment with human ApoE3 reversed the reduced inducibility of ApoE-free lipoproteins. Moreover, we observed that inhibition of Sp1 DNA-binding by mithramycin A diminished ABCA1 expression and ApoAI-mediated cholesterol efflux induced by ApoB-carrying lipoproteins, and that mutation of the Sp1-binding motif in the ABCA1 promoter region diminished ApoB-carrying lipoprotein-induced ABCA1 promoter activity. Collectively, these data suggest that ApoE associated with ApoB-carrying lipoproteins has an upregulatory role on ABCA1 expression, and that induction of Sp1 phosphorylation is a mechanism by which ApoE upregulates ABCA1 expression.

## Introduction

Apolipoprotein (Apo) E is a major component of ApoB-carrying lipoproteins chylomicron remnants and very-low density lipoprotein (VLDL). It was initially recognized for its receptor binding function. Under physiological conditions, ApoE, by binding to cell surface low-density lipoprotein receptor (LDLR) or LDLR-related protein (LRP), initiates receptor-mediated endocytosis [1]. Further studies reveal that ApoE has, among other functions, a role in cholesterol efflux. Two mechanisms currently have been suggested for ApoE-mediated cholesterol efflux. One of them involves lipoprotein-associated ApoE [2], [3]. Following receptor-mediated endocytosis, ApoB-carrying lipoproteins disintegrate in the early sorting endosomes. The lipid core and ApoB are directed into lysosomal compartments, while ApoE and receptors are recycled back to the plasma membrane, followed by ApoE resecretion [4]. ApoE recycling is accompanied with cholesterol efflux [2]. Impaired ApoE recycling has been shown to result in intracellular cholesterol accumulation [3]. Another mechanism for ApoE-mediated cholesterol efflux involves lipid-poor ApoE [5], [6]. Increasing evidence indicates that many extrahepatic cells, including macrophages, secret ApoE, which promotes macrophage cholesterol efflux by autocrine or paracrine. In this process, lipid-poor ApoE accepts cholesterol released from macrophages [5], [6], resulting in assembly of nascent high-density lipoproteins (HDL) [7], [8].

Despite much progress in research of ApoE-mediated cholesterol efflux, very little is known about the effect of ApoE on the expression of cholesterol transporters. ATP-binding cassette transporter A1 (ABCA1) is one of the plasma membrane proteins that export excess cholesterol [9]. Loading cells with cholesterol or oxysterol, or incubation with native or oxidized LDL has been shown to induce ABCA1 expression [10]–[14]. It has been suggested that increase in cellular cholesterol activates nuclear transcription factor liver X receptor (LXR), which in turn induces ABCA1 expression [15]. Two isoforms of LXR have been identified in mammalian cells. LXRα is primarily expressed in liver, kidney, macrophages and intestine, whereas LXRβ is ubiquitously expressed [16]. Oxysterols have been shown to function as ligands that bind and activate LXR [11]. The oxysterol-activated LXRs form heterodimers with retinoid X receptor (RXR) and bind to the ABCA1 promoter, inducing transcription [11], [15]. A recent study showed that treatment of cells with oxysterols induced physical interaction of specificity protein 1 (Sp1) with the LXR and RXR heterodimer in the ABCA1 promoter [17]. Inhibition of Sp1 DNA-binding attenuated oxysterol-induced ABCA1 expression [17]. These observations suggest that oxysterol-induced ABCA1 expression is modulated by Sp1. However, very little is known about how Sp1 is involved in regulation of ABCA1 expression.

The present report examined the effect of ApoE on ApoB-carrying lipoprotein-induced ABCA1 expression in macrophages. Our data demonstrate that ApoB-carrying lipoproteins from both wild-type and ApoE knockout mice induced ABCA1 expression and increased ABCA1 promoter activity, which was associated with an enhanced Sp1 phosphorylation and increased Sp1 binding to the ABCA1 promoter. The increased magnitude was significantly less in macrophages treated with ApoE-free lipoproteins than in cells treated with wild-type lipoproteins. ApoE enrichment reversed the inhibitory effects of the ApoE-free lipoproteins. These observations inform a novel function for ApoE, *i.e.*, ApoE associated with ApoB-carrying lipoprotein upregulates ABCA1 expression.

## Methods

### Animals

Wild-type C57BL/6 and ApoE knockout (*ApoE^−/−^*) mice were obtained from the Jackson laboratory (Bar Harbor, ME). The *ApoE^−/−^* mice were generated by Piedrahita *et al.* [18] and were backcrossed to C57BL/6 for over 10 generations. All mice at 3–4 months of age were fed a high-fat diet containing 15% fat and 1.25% cholesterol by weight (Harlan Teklad, Madison, WI) for 4 days. Blood (0.5–1 ml) was collected from the posterior vena cava after mice were anesthetized with 0.03 ml of cocktail containing 80 mg/ml ketamine hydrochloride and 12 mg/ml xylazine hydrochloride. To minimize oxidation, collected blood was immediately mixed with 50 µM butylated hydroxytoluene and 2 mM EDTA and cooled on ice. All procedures for handling animals were conducted following protocols approved by the Institutional Animal Care and Use Committee at Meharry Medical College (Protocol Number: 080530ZG124).

### Lipoprotein isolation

Lipoproteins were isolated from the plasma of wild-type and *ApoE* ^−/−^ mice, respectively. Mouse plasma mixed with 50 µM of butylated hydroxytoluene (BHT) was overlaid with a potassium bromide (KBr) gradient solution (*d*: 1.063) and centrifuged at 120,000 rpm for 2 hrs with a Sorvall Discovery M150 ultracentrifuge (Kendro Laboratory Products, Asheville, NC). The upper fraction containing ApoB-carrying lipoproteins was collected, and dialyzed in phosphate buffered saline (PBS, pH 7.4) containing 10 mM EDTA at 4°C for 48 hrs, and filtered through a 0.45-µm filter [19].

### ApoE enrichment

The ApoE-free, ApoB-carrying (E^−^/B) lipoproteins were enriched with human ApoE3 as described by Clavey et al. [20]. The ApoE/cholesterol ratio in the ApoB-carrying lipoproteins obtained from wild-type mice [21] and normolipidemic subjects [22], [23] is about 1–3∶100 (10–30 µg ApoE : 1 mg cholesterol). In this study, E^−^/B lipoproteins were incubated with human ApoE3 (Sigma-Aldrich, St. Louis, MO) at a ratio of 10 µg ApoE : 1 mg cholesterol at 37°C for 1 hr. The ApoE3-enriched, ApoB-carrying (E3/B) lipoproteins were re-isolated by KBr gradient ultracentrifugation as described above to remove free ApoE.

### Cholesterol efflux assay

Raw 264.7 cells (ATCC, Manassas, VA) grown in 24-well plates were incubated with 5 µCi/ml of [1,2-^3^H(N)]-cholesterol (Perkin Elmer, Waltham, MA) in the presence or absence of 20 µg/ml of E^+^/B, E^−^/B or E3/B lipoproteins for 24 hrs. After washed with PBS, cells were further incubated with 20 µg/ml of human ApoAI (Sigma-Aldrich) or culture medium. After a 2-hr incubation, the culture medium was collected. Cells were lysed with 0.5 M NaOH. The lysate and medium were mixed with scintillation fluid for radioactivity assay using a Tri-Carb 2300TR Liquid Scintillation Analyzer (Perkin Elmer). Cholesterol efflux was expressed as the percentage of radioactivity in medium to total radioactivity (cells plus medium) [9]. ApoAI-mediated cholesterol efflux was calculated as the difference between the values obtained in the presence or absence of ApoAI in the medium.

### Quantitative real-time RT-PCR analysis

Raw2 64.7 cells grown in six-well plates were treated with 20 µg/ml of E^+^/B, E^−^/B or E3/B lipoproteins or culture medium alone as a control for 4 hrs. Total RNA was extracted using a RNeasy Mini kit (Qiagen, Valencia, CA), and subjected to reverse transcription using PolyT and Superscript III First-Strand (Invitrogen, Carlsbad, CA). The resulting cDNAs were subjected to quantitative real-time PCR with an iCycler system (Bio-Bad, Hercules, CA) using primers synthesized by Qiagen (Valencia, CA) for amplification of ABCA1 and 3-phosphate dehydrogenase (GAPDH) [24]. The expression levels of the ABCA1 mRNAs were normalized to GAPDH mRNA.

### Western blot analysis

Raw 264.7 cells grown in 6-well plates were treated with 20 µg/ml of E^+^/B, E^−^/B or E3/B lipoproteins or culture medium alone for 4 hrs. Cells were lysed with M-PER reagent supplemented with protease inhibitor and a phosphatase cocktail [1 mM Na_3_VO_4_, 1 mM dithiothreitol (DTT), and 1 mM phenylmethylsulfonyl fluoride (PMSF)]. Protein concentrations in the lysates were determined using a BCA protein assay kit. Samples containing 40 µg of protein were subjected to on 6% (for separation of phosphorylated and non-phosphorylated Sp1) or 10% (for separation of other proteins) sodium dodecyl sulfate-polyacrylamide gel electrophoresis. Proteins were then transferred to polyvinylidene fluoride membranes. Antibodies against Sp1, ABCA1 and β-actin (Santa Cruz Biotechnology, Santa Cruz, CA), followed by detection with horseradish peroxidase-conjugated secondary antibodies (Santa Cruz Biotechnology), were used as described previously [25]. Immunoreactive bands were visualized using ECL-plus chemiluminescence reagent (GE Healthcare Healthcare–Amersham, Piscataway, NJ) and analyzed with a GS-700 Imaging Densitometer (Bio-Rad).

### Chromatin immunoprecipitation (ChIP) assay

Raw 264.7 cells were plated in 100-mm tissue culture dishes and treated with 20 µg/ml of E^+^/B, E^−^/B or E3/B lipoproteins or culture medium alone for 4 hrs. The amount of Sp1 bound to the ABCA1 promoter was determined by ChIP analysis as described previously [24]. Briefly, macrophages were cross-linked with 1% formaldehyde, and lysed in cell lysis buffer (5 mM PIPES, 85 mM KCl, 0.5% Nonidet P-40, 1 mM PMSF, and protease inhibitor cocktail, pH 8.0). Nuclei were isolated and homogenized in nuclear lysis buffer (50 mM Tris–HCl, 1% SDS, 10 mM EDTA, and protease inhibitor cocktail, pH 8.1), and then sonicated until crosslinked chromatin was sheared to an average length of 0.3∼1.0 kb. A 5 µl aliquot of supernatant was used as an input control. The remaining lysate was diluted 10-fold with ChIP dilution buffer (16.7 mM Tris–HCl, 167 mM NaCl, 0.01% SDS, 1.1% Triton X-100, 1.2 mM EDTA, and protease inhibitor cocktail, pH 8.1), and precleaned with salmon sperm DNA (Invitrogen, Carlsbad, CA) and protein A-agarose (Santa Cruz Biotechnology). The precleaned sample was incubated with Sp1 antibody, followed by salmon sperm DNA/protein A-agarose. Bound protein-DNA complexes were eluted with a solution containing 0.1 M NaHCO_3_ and 1% SDS. Following reversion of the protein-DNA crosslinks, DNA fragments in the eluate and input controls were purified using the QIAquick PCR purification kit (Qiagen), and then subjected to quantitative real-time PCR using an iCycler system (Bio-Rad). The PCR primers used were: forward 5′-dTTCCCGTTTCCCGAAGGCTA-3′ and reverse 5′-dAACGCTGTTCTCTCCCTCTT-3′. A 220-bp DNA fragment containing the Sp1-binding site sequence of the ABCA1 promoter region was amplified. The amount of DNA-bound Sp1 was expressed as a ratio of the PCR product amplified from the anti-Sp1 immunoprecipitants versus the input controls.

### Luciferase and β-galactosidase assays

For functional analysis of the ABCA1 promoter, two 5′-deletion and one mutant ABCA1 promoter fragments were generated by PCR using UltraPfu DNA Polymerase. For generation of the deletion fragments, wild-type C57BL/6J mouse genomic DNA was used as a template. The PCR reverse primer for these fragments starts at nucleotide 205 downstream from the transcription start site (TSS) of the ABCA1 gene. The forward primers start at nucleotides −1094 or −125 upstream from the TSS of the ABCA1 gene for generation of a 1299-bp and a 330-bp PCR fragment, respectively. The shorter PCR fragment, referred to as ABC-125, was used as a template to generate a mutant ABCA1 promoter fragment, in which the Sp1-binding site was mutated. The PCR products were cloned into the *Kpn*I-*Xho*I sites of the pGL2 luciferase vector (Promega, Madison, WI). The recombinant plasmid construct (or empty pGL-2 vector) and a β-galactosidase expression plasmid (pCMV-SPORT-βgal, Promega) were co-transfected into RAW264.7 cells using a Nucleofector II as described previously [26]. The transfected cells were treated with 20 µg/ml of E^+^/B, E^−^/B or E3/B lipoproteins or culture medium alone for 4 hrs, and lysed with 100 µl of lysis buffer provided by the luciferase assay kit or the galactosidase assay kit (Galacto-Star™ ß-Galactosidase Reporter Gene Assay System). A 10 µl aliquot of lysate was incubated in a 96-well plate at room temperature for 2 min with 100 µl of luciferase assay reagent (Promega) or for 60 min with 100 µl of galactosidase assay reagent. Luminescence was measured using the BL10000 LumiCount (Packard BioScience, Meriden, CT). Luciferase activity was expressed as the luminosity ratio of the luciferase assay to the β-galactosidase assay [25].

### Statistical analysis

Data were reported as mean ± standard error of the mean. Differences among cells treated with E^+^/B, E^−^/B, E3/B lipoproteins or culture medium alone were analyzed by one-way analysis of variance (ANOVA) and the Student's unpaired *t*-test, unless otherwise indicated. Statistical significance was considered when *P* was less than 0.05. Statistix software (Statistix, Tallahassee, FL) was used for statistical analysis. The number of experiments performed is indicated in figure legends.

## Results

### The effect of ApoE on ApoB-carrying lipoprotein-induced ABCA1 expression and cholesterol efflux

**Figure 1:**
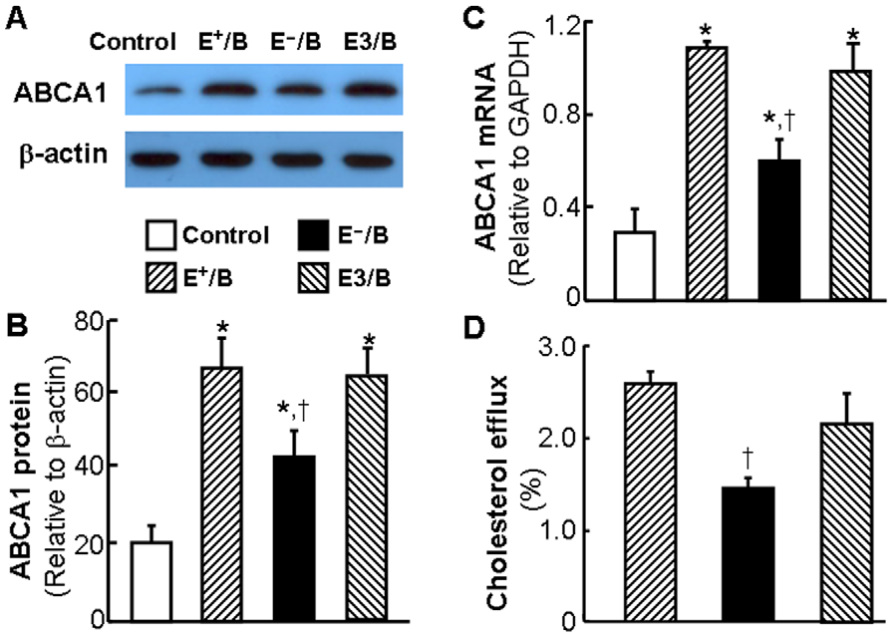
The effect of ApoE on lipoprotein-induced ABCA1 expression and cholesterol efflux. For study the effect of ApoB-carrying lipoproteins on ABCA1 expression, mouse macrophages were incubated with 20 µg/ml of wild-type (E^+^/B), ApoE-free (EÎ/B) or ApoE3-enriched (E3/B) lipoproteins, or culture medium alone. **Panel A and B:** The level of ABCA1 protein was determined by western blot analysis and quantitated relative to β-actin. **Panel C:** The level of ABCA1 mRNA was determined by quantitative real-time RT-PCR and normalized to GAPDH mRNA. **Panel D:** For studying the effect of ApoE on lipoprotein-induced cholesterol efflux, mouse macrophages were incubated with [^3^H]-cholesterol in the presence or absence of 20 µg/ml of E^+^/B, E^−^/B or E3/B lipoproteins. ApoAI-mediated cholesterol efflux was measured as described under Materials and Methods. Values represent the mean ± SEM of five independent experiments. Differences among cells treated with E^+^/B, E^−^/B, E3/B lipoproteins or culture medium alone were analyzed by one-way ANOVA followed by Tukey post-hoc tests (B and C) or Student *t*-test (D). * *P*<0.05 compared to control, ^†^ *P*<0.05 compared to E^+^/B or E3/B lipoprotein treatment.

In this study, we first examined the effect of ApoB-carrying lipoproteins on the expression of macrophage ABCA1, a plasma membrane protein that transports cholesterol from cell membrane to lipid-free ApoAI [27]. As shown in Fig. 1A–1B, the basal ABCA1 protein level was relatively low. Treatment of macrophages with 20 µg/ml of E^+^/B lipoproteins for 4 hrs elevated the ABCA1 protein level by more than 3.2 fold (Fig. 1A–1B), which was associated with a 3.7-fold increase in ABCA1 mRNA level (Fig. 1C). In contrast, the same dose of E^−^/B lipoproteins resulted in only approximately 2-fold increase in ABCA1 mRNA and protein. Enrichment with ApoE increased the ability of E^−^/B lipoproteins to induce ABCA1 expression. The level of ABCA1 mRNA and protein induced by E3/B lipoproteins was comparable to that induced by E^+^/B lipoproteins (Fig. 1A–1C). These observations suggest that ApoE is indispensible for a full induction of ABCA1 expression by ApoB-carrying lipoproteins, *i.e.*, ApoE has an upregulatory role in ApoB-carrying lipoprotein-induced ABCA1 expression.

Having demonstrated the difference of ABCA1 expression induced by ApoB lipoproteins in the presence or absence of ApoE, we then studied the effect of these lipoproteins on ApoAI-induced cholesterol efflux. Data in Fig. 1D show that treatment of macrophages with either E^+^/B or E^−^/B lipoproteins enhanced ApoAI-mediated cholesterol efflux. However, the enhancement was significantly less in cells treated with E^−^/B lipoproteins than in those treated with E^+^/B lipoproteins (2.82±1.13% versus 1.11±0.11%). The data in Fig. 1D also show that E3/B lipoproteins induced similar amount of cholesterol efflux as E^+^/B lipoproteins. These findings imply that induction of ABCA1 expression might be one of the mechanisms by which ApoE promotes cholesterol efflux.

### The effect of ApoE on ApoB-carrying lipoprotein-induced ABCA1 promoter activity

**Figure 2:**
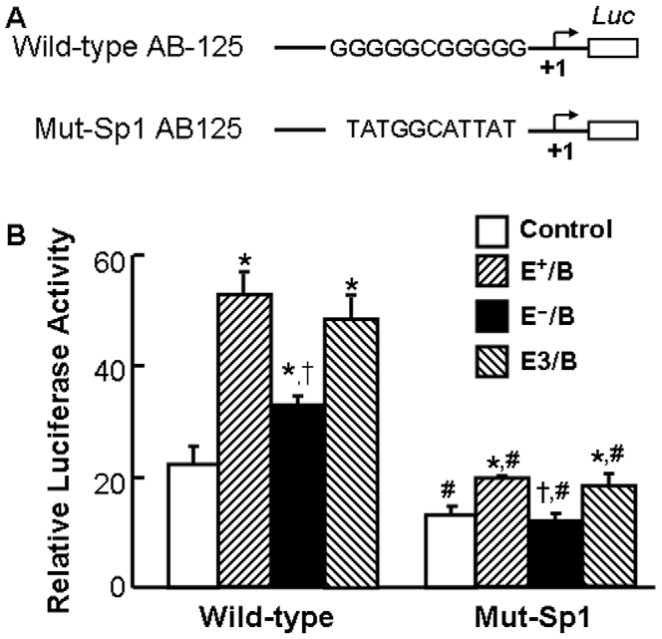
The effect of ApoE on lipoprotein-induced ABCA1 promoter activity. **Pane A:** Two constructs containing wild-type and Sp1-binding site-mutated (Mut-Sp1) ABCA1 promoter region were subcloned into a luciferase (Luc) reporter plasmid (pGL-2). The mutated sequences in the Mut-Sp1 construct are shown in the schematic diagram. **Panel B:** Mouse macrophages were transfected with a β-galactosidase expression plasmid and the ABCA1 promoter-reporter constructs or the empty pGL-2 vector. The transfected macrophages were treated with 20 µg/ml of wild-type (E^+^/B), ApoE-free (EÎ/B) or ApoE3-enriched (E3/B) lipoproteins, or culture medium alone (control) for 4 hrs. Luciferase activity was normalized to the β-galactosidase activity and expressed relative to that of pGL2 basic vector. Values represent the mean ± SEM of five independent experiments. * *P*<0.05 compared to cells transfected with the same plasmid and without lipoprotein treatment (control), ^†^ *P*<0.05 compared to cells transfected with the same plasmid and treated with E^+^/B or E3/B lipoproteins, and ^#^ *P*<0.05 compared to cells transfected with wild-type ABCA1 promoter and treated with the same lipoproteins.

In this study, we initially examined the effect of E^+^/B lipoproteins on ABCA1 promoter activity by transfection of macrophages with two ABCA1 promoter-luciferase reporter chimeric constructs. One contains 1094 bp, and the other contains 125 bp of the ABCA1 promoter. We observed that the basal and lipoprotein-induced promoter activities in these two fragments were comparable (data not shown). We then compared the effect of E^+^/B, E^−^/B and E3/B lipoproteins on ABCA1 promoter activity using the promoter-luciferase reporter construct containing 125 bp of the ABCA1 promoter (ABC-125). The data in Fig. 2 show that treatment of macrophages with E^+^/B, E^−^/B or E3/B lipoproteins induced about 2.4-, 1.5- and 2.2-fold elevation in luciferase activity, respectively. The E^−^/B lipoprotein-induced luciferase activity was significantly lower than that induced by E^+^/B or E3/B lipoproteins. These data suggest that increasing ABCA1 promoter activity is a mechanism by which ApoB-carrying lipoproteins induce ABCA1 expression, and that ApoE enhances the regulatory role of ApoB-carrying lipoproteins on ABCA1 promoter activity.

It has been demonstrated Sp1 is a transcription factor that controls ABCA1 expression [15], [17]. Computer analysis of the ABCA1 promoter shows a Sp1-binding site located 99 nucleotides upstream of the TSS. As the data in Fig. 2 show, mutation of this Sp1-binding motif significantly reduced the basal and lipoprotein-induced expression of the reporter gene. Specifically, the basal luciferase activity was about 40% lower in macrophages transfected with the Sp1 motif-mutated ABCA1 promoter than in those transfected with the wild-type ABCA1 promoter counterpart (Fig. 2). Treatment with E^+^/B or E3/B lipoproteins induced only about 1.5-fold elevation in luciferase activity in macrophages transfected with the mutated ABCA1 promoter, while E^−^/B lipoproteins did not induce any luciferase activity in these cells. These inductions were significantly less than lipoprotein-induced luciferase activities in cells transfected with the wild-type ABCA1 promoter. Taken together, the Sp1binding motif is essential for basal and lipoprotein-induced ABCA1 promoter activities in the presence or absence of ApoE.

### The effect of ApoE on ApoB-carrying lipoprotein-induced Sp1 binding to the ABCA1 promoter

**Figure 3:**
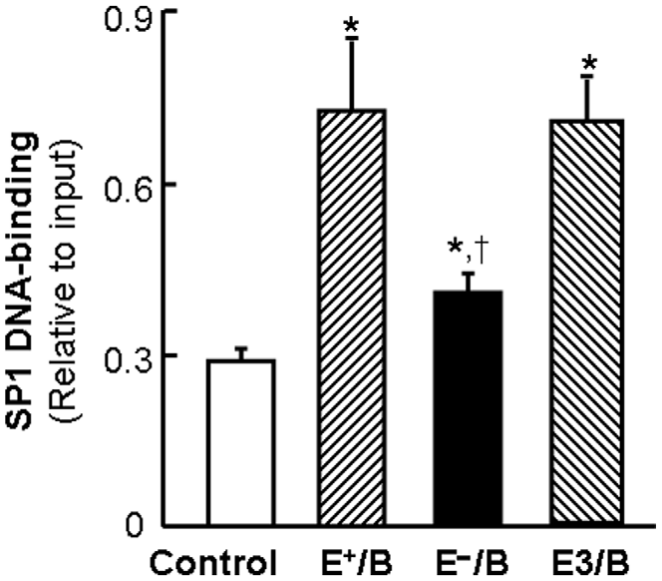
The effect of ApoE on lipoprotein-induced Sp1 DNA-binding activity. Mouse macrophages were treated with 20 µg/ml of wild-type (E^+^/B), ApoE-free (EÎ/B) or ApoE3-enriched (E3/B) lipoproteins, or culture medium alone (control) for 4 hrs. The amount of Sp1 bound to the ABCA1 promoter region was determined by ChIP analysis, and expressed as the ratio of input controls. Values represent the mean ± SEM of 5 separate experiments. * *P*<0.05 compared to control, ^†^ *P*<0.05 compared to E^+^/B or E3/B lipoprotein treatment.

To confirm the regulatory role of Sp1 in lipoprotein-induced ABCA1 promoter activity, we determined the amount of Sp1 bound to the ABCA1 promoter in macrophages treated with E^+^/B, E^−^/B and E3/B lipoproteins. As the data in Fig. 3 show, the ability of ApoB-carrying lipoproteins to induce Sp1 binding to the ABCA1 promoter region varied in the presence or absence of ApoE. Specifically, the amount of Sp1 proteins bound to the ABCA1 promoter was increased about 2.5 fold in macrophages treated with E^+^/B and E3/B lipoproteins, whereas the DNA-bound Sp1 was increased only about 1.4-fold in cells treated with E^−^/B lipoproteins (Fig. 3). These results suggest that ApoE enhances the capability of ApoB-carrying lipoproteins to induce Sp1 binding to the ABCA1 promoter region.

### The effect of ApoE on ApoB-carrying lipoprotein-induced Sp1 expression and phosphorylation

**Figure 4:**
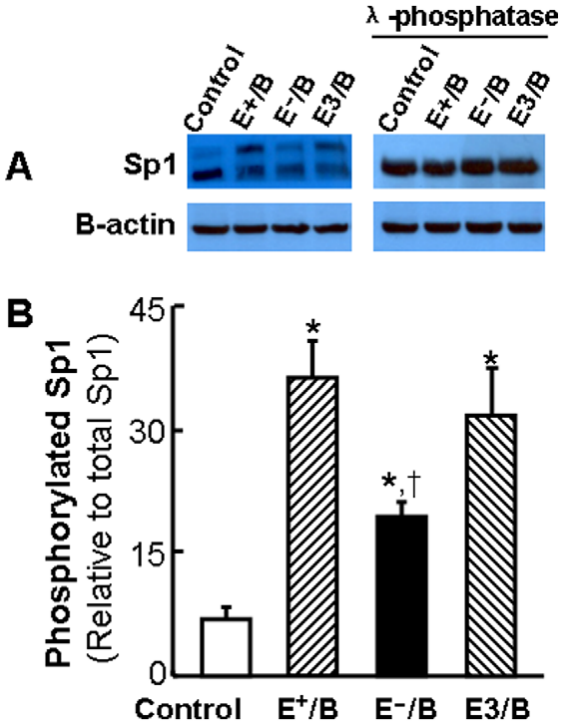
The effect of ApoE on lipoprotein-induced Sp1 phosphorylation. Mouse macrophages were treated with 20 µg/ml of wild-type (E^+^/B), ApoE-free (EÎ/B) or ApoE3-enriched (E3/B) lipoproteins, or culture medium alone (control) for 4 hrs. The cell lysate were incubated with or without λ-phosphatase. Sp1 phosphorylation was detected by Sp1 immunoblot band shift induced by lipoprotein and λ-phosphatase treatments. The level of phosphorylated Sp1 was expressed as the percentage of the immunoreactive intensity of the top band versus the total (top plus bottom bands). Values represent the mean ± SEM of 3 separate experiments. * *P*<0.05 compared to control, ^†^ *P*<0.05 compared to E^+^ or E3 lipoprotein treatment.

To uncover the mechanism underlying the induction of Sp1 binding to the ABCA1 promoter by ApoB-carrying lipoproteins, we examined the total and phosphorylated Sp1 protein levels. As the typical western blot images in Fig. 4A illustrate, two Sp1 immunoreactive bands were detectable, indicating covalent modifications in Sp1 proteins. While ApoB-carrying lipoprotein treatment did not significantly alter the total Sp1 protein level, a band shift was visualized in cells treated with lipoproteins (Fig. 4A). Specifically, ApoB-carrying lipoproteins induced part of Sp1 proteins shifting to the top band. To determine whether the slow migrating forms of Sp1 were generated by phosphorylation, we incubated an aliquot of the cell lysate with λ-phosphatase before western blot analysis. As shown in Fig. 4A, λ-phosphatase abolished the slowly migrating form of Sp1 induced by ApoB-carrying lipoproteins. These observations indicate that the lipoprotein-induced shift of the Sp1 inmmunoreactive band is due to Sp1 phosphorylation. The data in Fig. 4B show that the level of Sp1 phosphorylation induced by E^−^/B lipoproteins was significantly reduced as compared to that induced byE^+^/B and E3/B lipoproteins. Increase in phosphorylation has been suggested to enhance the activity of Sp1 to bind its cognate DNA sequences [28]. Taken together, our data suggest that induction of Sp1 phosphorylation might be a mechanism by which ApoB-carrying lipoproteins induce Sp1 binding to the ABCA1 promoter, and that ApoE enhances the ability of ApoB-carrying lipoproteins to induce Sp1 phosphorylation.

### Mithramycin A inhibits ApoB-carrying lipoprotein-induced ABCA1 expression and cholesterol efflux

**Figure 5:**
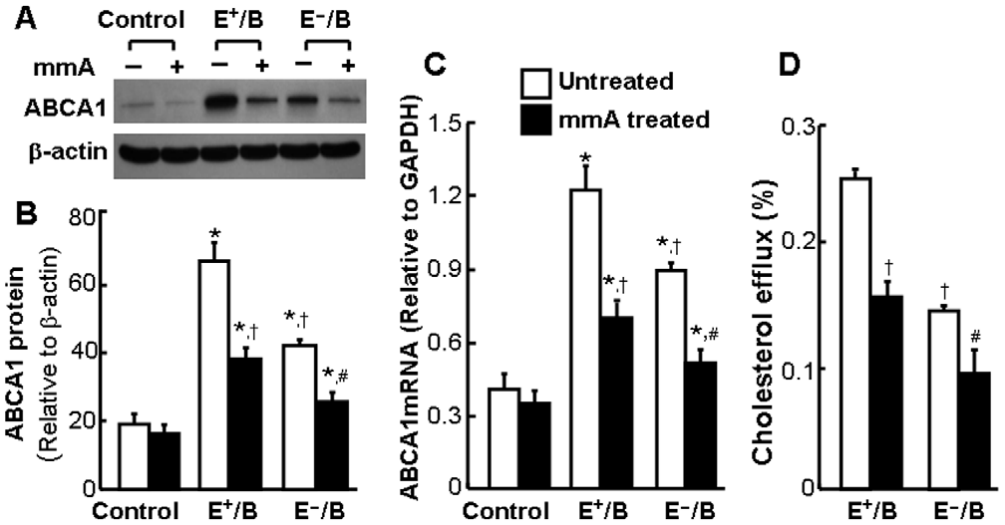
The effect of mithramycin A on lipoprotein-induced ABCA1 expression and cholesterol efflux. For study ABCA1 expression, mouse macrophages were treated with 20 µg/ml of wild-type (E^+^/B), ApoE-free (EÎ/B) or ApoE3-enriched (E3/B) lipoproteins, or culture medium (control) in the presence or absence of 100 nM mithramycin A (mmA) for 4 hrs. **Panel A and B:** The level of ABCA1 protein was determined by western blot analysis and quantitated relative to β-actin. **Panel C:** The level of ABCA1 mRNA was determined by quantitative real-time RT-PCR and normalized to GAPDH mRNA. **Panel D:** For studying cholesterol efflux, mouse macrophages were incubated with [^3^H]-cholesterol in the presence or absence of 20 µg/ml of E^+^/B, E^−^/B or E3/B lipoproteins for 24 hrs. ApoAI-mediated cholesterol efflux was measured in the presence or absence of mmA. Values represent the mean ± SEM of five independent experiments. * *P*<0.05 compared to control, ^†^ *P*<0.05 compared to cells treated with E^+^/B lipoproteins and without mmA, and ^#^ *P*<0.05 compared to cells treated with E^−^/B lipoproteins and without mmA.

To address whether Sp1 binding to ABCA1 promoter is necessary for ApoB-carrying lipoprotein-induced expression of ABCA1, we studied the effect of mithramycin A on ABCA1 expression. Mithramycin A is a chemotherapeutic drug that binds to GC-rich DNA sequences thereby blocking the binding of Sp1 to GC-specific regions of DNA [29]. A wide range of concentrations of mithramycin A (10 nM–1 µM) have been used to inhibit the transcriptional factor activity of Sp1 in various types of cells, including human bronchial epithelial cells, multiple myeloma cells [29], [30], and mouse endothelial cells [25]. As the data Fig. 5A–5C show, addition of 100 nM mithramycin A significantly diminished ABCA1 mRNA and protein expression induced by E^+^/B and E^−^/B lipoproteins. These data suggest that binding of Sp1 protein to the ABCA1 promoter is indispensible to ABCA1 expression induced by ApoB-carrying lipoproteins in the presence or absence of ApoE. In addition, inhibition of ABCA1 expression by mithramycin A was associated with a reduced cholesterol efflux (Fig. 5D), suggesting that induction of ABCA1 expression is a mechanism for ApoB-carrying lipoprotein-induced cholesterol efflux.

## Discussion

Using ApoB-carrying lipoproteins obtained from ApoE knockout mice, this report demonstrates that lipoprotein-associated ApoE has an upregulatory role on ABCA1 expression. In the absence of ApoE, ApoB-carrying lipoproteins manifested reduced ability to increase ABCA1 mRNA and protein in macrophages, when compared to wild-type lipoproteins. Enrichment of ApoE-free lipoproteins with ApoE3, the so-called normal isoform of human ApoE, increased ABCA1 expression to the level similar to that induced by wild-type lipoproteins. In agreement with the ABCA1 expression level, the ApoAI-mediated cholesterol efflux was significantly lower in macrophages treated with ApoE-free lipoproteins than in those treated with ApoE-containing lipoproteins. It has been well-established that lipid-free ApoAI serves as a cholesterol acceptor for ABCA1-mediatd cholesterol efflux [27]. Taken together, upregulation of ABCA1 expression may be one of the mechanisms by which ApoE increases macrophage cholesterol efflux. ApoE has been shown to regulate cholesterol efflux by its recycling [2] and by functioning as a cholesterol acceptor [5], [6].

Emerging evidence indicates that ABCA1 expression is tightly regulated at the transcription level by cellular cholesterol content [31]. Cholesterol-induced ABCA1 expression is attributed to nuclear transcription factor LXR [10], [31]. Paradoxically, a previous work from our laboratory showed that the cholesterol content was significantly higher in macrophages treated with ApoB-carrying lipoproteins obtained from ApoE knockout mice than in those treated with lipoproteins obtained from wild-type mice [19]. In addition, the level of oxidized lipids was significantly higher in ApoE-free lipoproteins than in wild-type lipoproteins [19]. Although these data do not exclude the participation of cholesterol-activated LXR in ApoB-carrying lipoprotein-induced ABCA1 expression, they do suggest that mechanisms other than increase in cell cholesterol level are responsible for the upregulatory role of ApoE on ABCA1 expression.

Data from this report clearly indicate that increase in the transcriptional activity of Sp1 is a mechanism underlying the upregulatory role of ApoB-carrying lipoproteins on ABCA1 expression. It is known that Sp1 regulates basal and inducible transcription of many genes via binding to their promoter region [32]. Mouse ABCA1 promoter contains one Sp1-binding site located 99 nucleotides upstream the transcription start site. Our data demonstrate that mutation of this DNA regulatory element weakened the ABCA1 promoter activity induced by ApoB-carrying lipoprotein. Inhibition of Sp1 DNA binding diminished ABCA1 expression induced by ApoB-carrying lipoproteins. These findings suggest that increasing Sp1 binding to its cognate DNA sequence in the ABCA1 promoter region is a mechanism for ApoB-carrying lipoprotein-induced ABCA1 transcription. Indeed, treatment of macrophages with ApoB-carrying lipoproteins increased Sp1 binding to the ABCA1 promoter region. Our data also demonstrate that ApoB-carrying lipoproteins devoid of ApoE displayed reduced ability to induce Sp1-ABCA1 promoter binding and reduced ABCA1 promoter activity, when compared to ApoE-containing lipoproteins. It appears that ApoE is indispensible for efficient induction of Sp1 binding to the ABCA1 promoter by ApoB-carrying lipoproteins.

Modification through phosphorylation has been suggested to increase the DNA-binding activity of Sp1 [28]. Our data demonstrate that ApoB-carrying lipoproteins induced Sp1 phosphorylation in mouse macrophages. Deficiency of ApoE diminished the inducibility of ApoB-carrying lipoproteins on Sp1 phosphorylation, which is consistent with the reduced ABCA1 promoter activity and ABCA1 expression induced by ApoE-free lipoproteins. These findings suggest a role of ApoE in activation of protein kinase(s) for Sp1 phosphorylation. Several kinases, including DNA-dependent protein kinase, protein kinase C-ζ (PKC- ζ), phosphatidylinositol 3-kinase (PI3K), casein kinase II, extracellular signal-regulated kinase (ERK), and cyclin-dependent kinase 2 (Cdk2), have been shown to mediate Sp1 phosphorylation [17]. Although the kinase(s) responsible for ApoE-induced Sp1 phosphorylation have not been defined, available evidence supports the view that ApoE is able to induce signaling transduction. It is known that ApoB-carrying lipoprotein-associated ApoE has high affinity for several members of the LDLR family, including LDLR, VLDL receptor (VLDLR), LRP1, LRP2, and LRP8 (ApoE receptor 2) [33]. While the role of the LDLR is limited to the regulation of cholesterol homeostasis by receptor-mediated endocytosis of lipoprotein particles, the other members of the gene family have additional physiological functions as signal transducers [33]. Thus, binding and internalization of ApoE-carrying lipoproteins might activate various signaling pathways, some of which may activate the protein kinase(s) that phosphorylate Sp1. For example, activation of VLDLR and LRP8 has been shown to activate PI3K [34]. In addition, activation of LRP1 has been shown to activate Ras, which has a function to activate PKC- ζ [35].

It is of interest that ApoB-carrying lipoproteins in the absence of ApoE can also induce Sp1 phosphorylation, despite smaller magnitude than in the presence of ApoE. These findings suggest that other component(s) in the ApoB-carrying lipoproteins besides ApoE can activate signaling pathway(s) involving protein kinase(s) for Sp1 phosphorylation. It has been suggested that macrophages are able to take up ApoB-carrying lipoproteins via scavenger receptor (SR)-mediated pathways in the absence of ApoE [36], [37]. Some SRs, such as SR-A, CD36 and LOX-1, have been implicated to initiate intracellular signaling cascades [38], [39]. It is highly likely that the ApoE-independent pathway(s) also activate protein kinase(s) that phosphorylate Sp1, inducing ABCA1 expression.

In summary, data from this report demonstrate that ApoB-carrying lipoproteins, in the presence or absence of ApoE, are able to induce macrophage ABCA1 expression via activation of transcription factor Sp1. The magnitude of induction is significantly greater in the presence of ApoE. These findings imply that there exist two distinct pathways for induction of Sp1 phosphorylation by ApoB-carrying lipoproteins, one depends on ApoE, and the other does not. Under physiological conditions, ApoB-carrying lipoproteins are internalized via ApoE-mediated pathways. The ApoE-dependent Sp1 phosphorylation and ABCA1 induction thus could be physiologically important for efflux of excess cholesterol derived from ApoB-carrying lipoproteins.
